# Moving Beyond Single Racial Identification: Considering Multiracial and Multiethnic Identification in Public Health

**DOI:** 10.1177/15248399241248409

**Published:** 2024-04-24

**Authors:** Tessa R. Pulido

**Affiliations:** 1 University of California, Irvine, Irvine, CA, USA

**Keywords:** health equity, advocacy, cultural competence, minority health, social determinants of health

## Abstract

As multiracial and multiethnic youth populations are anticipated to be 11.3% of the U.S. population by 2060, it is essential that public health research and practice find ways to effectively capture and reach these diverse groups. Single racial identification has been a norm in public health practice; however, this method has limitations for capturing the health of multiracial and multiethnic individuals. Drawing on personal experience of the author and multidisciplinary scholarship, this research commentary examines the limitations of single race identification and how this influences the processes of racialization. The author provides important implications for public health research by suggesting more complex and effective ways to capture personal racial identification and racial perceptions and addresses how to reach multiracial and multiethnic groups through public health interventions where individuals might identify with multiple cultural identities.

For most of my adolescence and young adulthood, I struggled to define and affirm my racial and ethnic identity. When I complete sociodemographic sections of surveys, I constantly feel unsure as to how to select from discrete racial categories. In elementary school, I could only choose one and usually alternated between “White” and “Asian.” I do not “look” White so, sometimes, I would consider my darker skin tone and how *others* perceived me and select “Asian.” Today, when presented with singular choices for race or ethnicity, I either opt for “other” or choose not to answer.

Response categories have expanded to include “two or more races” or “multiracial,” which leads me to reflect on what these options really mean in the context of public health. Public health researchers and practitioners often use race and ethnicity data in their analyses, as variables to describe health outcomes or disparities or in models to predict risk or health outcomes. However, for the aforementioned reasons, using a singular multiracial category can be problematic and raises concerns about the data’s accuracy or its representative nature for multiracial individuals.

Moving beyond single racial identification in public health is essential—by 2060, it is estimated that 11.3% of the U.S. population under 18 will identify as multiracial (Atkin, Christophe, et al., 2022). What can we learn about multiracial people and their health risk factors by using discrete racial categories, when every possible racial combination can exist under the term “multiracial?” How is this a unifying cultural construct and what does it represent? While using “select all that apply” is an improvement that captures unique racial combinations, it raises its own set of difficulties.

Collectively, these issues and questions highlight the importance of prioritizing *how to better understand*, *conduct research with*, and *serve multiracial and multiethnic individuals and groups to advance population health.*

Grappling with these challenges now will help us improve how race data are collected and used in public health research and practice. Racial classification operates as a socially constructed political system and may contribute to worse health outcomes for minoritized populations. Racial hierarchies establish belonging through physical characteristics, and multiracial people may pose a direct challenge to this type of classification (Haskell, 2022). In some cases, asking multiracial individuals to select a single racial category or be assigned one can be discriminatory and contribute to stress (Tabb, 2016). While there is an abundance of evidence that race is important in public health research and that racism impacts health outcomes, what do we know about the health of individuals who exist in and across multiple ethnic and racial categories (Bailey et al., 2017)? We should promote research to critically examine what racial and ethnic identities and identity perceptions should be captured in close-ended survey questions and use qualitative methods, particularly cognitive interviewing techniques, to populate these items.

Furthermore, what do we know about individuals who identify as multiracial? Using National Longitudinal Study of Adolescent to Adult Health (ADD Health) waves I and III data, multiracial young adults who engaged in racial category switching were more likely to have better self-reported health, which might be attributed to their ability to adapt to different environments and contexts known as protean identity (Tabb, 2016). Importantly, Tabb (2016) notes an individual’s racial identification may occur simultaneous to personal categorization, and identity formation occurs as multiracial and multiethnic individuals explicitly state their identity while answering surveys with existing categories. In short, asking people to identify their race *shapes* their racial identity. Researchers and practitioners should take note since many may be unaware of this socially dynamic impact on some respondents when they elect to include this question in data collection.

Multiracial identity formation is also heavily influenced by environmental, social, and political contexts. Racialized experiences impact how a multiracial person identifies and how they are perceived (Rockquemore et al., 2009). In another ADD Health study exploring differences in racial identity changes, misidentification of one’s racial group was associated with lower mental health outcomes, which might be indicative of life experiences encountering discriminatory practices and attitudes (Haskell, 2022). Understanding the differences between identity and perceived identity in public health has implications for interventions and health care delivery, because racialization occurs through actors within systems and structures operating on perceived identity instead of a multiracial person’s chosen identity (Atkin, Jackson, et al., 2022). Sociologists continue to emphasize the need for research on experiences and outcomes of multiracial children, including utilizing theories of critical race theory and colorism with a social-ecological approach to understand how multiracial identity formation impacts experiences from interpersonal to institutional levels (Burton et al., 2010). This approach would also be highly beneficial to integrate into public health training and scholarship to strengthen traditional disciplines like epidemiology, biostatistics, health behavior, and health services research.

Developing and evaluating multicultural interventions in public health practice with these considerations in mind can lead to innovative and transformative outcomes for this growing segment of the population. Evidence suggests individuals with stronger ties to cultural practices of their racial/ethnic group can lead to improved health and psychosocial outcomes. A study conducted with a sample of Filipino Americans (*n* = 2,000) revealed those with stronger ties to cultural practices experienced less depressive episodes compared with their counterparts (Mossakowski, 2003). Stronger racial/ethnic identification with one’s group also serves as a buffer toward experiencing depressive episodes when faced with experiences of racial/ethnic discrimination (Mossakowski, 2003). A review article of minoritized adolescents and their psychosocial, academic, and health risk behavior outcomes found that general positive feelings about one’s ethnic or racial identity was associated with better psychosocial adjustment for Black and Latinx youth, and better academic outcomes for Black, Latinx, Asian American and Pacific Islander (AAPI) youth (Rivas-Drake et al., 2014). In addition, multiracial and multiethnic individuals may reflect flexibility and connect with multiple cultures or respond to messaging tailored to different cultural groups they claim. Engaging their right to claim that group can improve their response to the intervention (Grilo et al., 2023; Rivas-Drake et al., 2014).

Researchers should shift toward analyzing both multiracial and ethnic identities along with a person’s identity perceptions. Grilo et al. (2023) synthesize a valuable approach to think about race data collection and how race is operationalized: Did the participant give their self-perceived race, was race collected by others, or was it based on discrete categories? Rockquemore et al. (2009) suggest researchers interested in larger scale racial patterns incorporate both racial identity and identification as separate variables, allowing for analyses using discrete racial categories (identification) and more nuanced data on racial identity.

Finally, understanding how racialization is experienced by multiracial people can help to reveal its influence on health outcomes. Due to my own experiences with racism and identity marginalization, I spent a lot of time internally screening my identity or worrying about how my identity might be perceived. I urge public health scholars to draw from sociology scholarship to explore identity formation and nuanced approaches to assess racial and ethnic identity. The limitations of discrete racial categorization are well-documented. As public health professionals, we have both an opportunity and a responsibility to critically evaluate measures to improve how we assess the health outcomes of multiracial and multiethnic people.
